# The British Medical Association

**Published:** 1901-07-13

**Authors:** 


### THE BRITISH MEDICAL ASSOCIATION.

At the coming meeting of the British Medical Association, which will be held at Cheltenham on July 30th to August 2nd, the address in medicine will be delivered by Dr. Goodhart, consulting physician to Guy's Hospital, and that in surgery by Sir William Thomson, C.B., surgeon to the Richmond Surgical Hospital, Dublin. There will also be illuminated fêtes, cinematographic illustrations of operations by Dr. Doyen, a soirée "followed by dancing," various excursions, the usual garden parties, the usual dinners, and the usual "sections." We were on the point of adding that the usual subjects would be discussed, but lest we should seem to be irreverent we will say that among the subjects to be discussed in the "sections" are chronic joint disease, neuritis in beer-drinkers, renal tension, gastro-jejunostomy, massage in injuries of joints, the causation, etc., of miscarriage, the treatment of metritis, enteric fever in its public health aspects, the *rôle* of toxic action in the pathogenesis of insanity, feeble-minded children, intussusception, nasal obstruction, malaria of course, organisation of military medical services, x-rays, light treatment, and a multitude of others; in fact, quite a representative stock-taking of all that we have been reading about for the last year or two. Evidently it will be a good meeting.

